# Prevalence of Human Papillomavirus Genotypes in Unvaccinated 16- to 20-Year-Old Men in Quebec, Canada

**DOI:** 10.1093/infdis/jiaf094

**Published:** 2025-02-21

**Authors:** Catherine Wolfe, Iulia Gabriela Ionescu, Marie-Hélène Mayrand, François Coutlée, Chantal Sauvageau

**Affiliations:** Département de médecine sociale et préventive, Faculté de médecine, Université Laval, Québec, Québec, Canada; Direction des risques biologiques-immunisation, Institut national de santé publique du Québec, Québec, Québec, Canada; Département d’obstétrique-gynécologie, Université de Montréal, Montréal, Québec, Canada; Axe carrefour de l'innovation, Centre de Recherche du Centre Hospitalier de l’Université de Montréal, Montréal, Québec, Canada; Axe immunopathologie, Centre de Recherche du Centre Hospitalier de l'Université de Montréal, Montréal, Québec, Canada; Départements clinique de médecine de laboratoire et de médecine, Services de biologie moléculaire et d’infectiologie, Centre Hospitalier de l’Université de Montréal, Montréal, Québec, Canada; Département de microbiologie, infectiologie et immunologie, Université de Montréal, Montréal, Québec, Canada; Département de médecine sociale et préventive, Faculté de médecine, Université Laval, Québec, Québec, Canada; Direction des risques biologiques-immunisation, Institut national de santé publique du Québec, Québec, Québec, Canada; Axe maladies infectieuses et immunitaires, Centre de Recherche du Centre Hospitalier Universitaire de Québec-Université Laval, Québec, Québec, Canada

**Keywords:** human papillomavirus (HPV), males, vaccination, prevalence, herd immunity

## Abstract

**Background:**

Since 2008, Quebec (Canada) has had a school-based human papillomavirus (HPV) vaccination program achieving >90% coverage (≥ 1 dose) in girls by age 15. In 2018, Quebec was the first jurisdiction to switch to a mixed schedule (nonavalent + bivalent), and as such wanted to evaluate it. However, when devising an evaluation strategy for new vaccine schedules, the presence of herd effect needs to be ascertained. With this in mind, this study aimed to measure HPV prevalence among unvaccinated 16- to 20-year-old sexually active men.

**Methods:**

In 2020–2022, men were recruited from schools and online across the province of Quebec. Participants completed an online questionnaire and provided a self-collected penile swab (surface) for HPV detection and genotyping (Anyplex II-HPV28 Detection assay). Risk factors associated with HPV positivity were assessed. Vaccination status (unvaccinated) was verified through the Quebec Vaccination Registry.

**Results:**

Overall, 369 participants provided a sample suitable for HPV testing. HPV prevalence was 18.4% (95% confidence interval [CI], 14.6%–22.8%). Only 2 participants harbored a quadrivalent (4vHPV)-targeted genotype (0.5% [95% CI, .1%–1.9%]), both of whom reported sexual contact with men. In multivariate analysis, age, greater number of lifetime sexual partners, and history of other sexually transmitted infections were independently associated with positivity for at least 1 HPV genotype.

**Conclusions:**

The low 4vHPV-targeted genotype prevalence (<1%) among unvaccinated men of the same age as women vaccinated with 4vHPV suggests a strong herd immunity among young adults in Quebec. Evaluation of schedule changes will have to take this finding into account.

**Clinical Trials Registration.** NCT04297670.

## Teaser

The very low prevalence of the quadrivalent human papillomavirus (HPV) vaccine–targeted genotypes among the unvaccinated male population of the same age as women who were eligible for vaccination suggests a strong herd immunity. This study shows the population effectiveness of HPV vaccination.

**(See the Editorial Commentary by Chesson and Markowitz on pages e189–92.)**

In 2008, a school-based human papillomavirus (HPV) vaccination program was implemented in the province of Quebec, Canada, initially targeting girls aged 9–17 years, who were primarily vaccinated in grade 4 with 2 doses of quadrivalent HPV (4vHPV) vaccine, with a 5-year catch-up program for those in grade 9 [1]. In 2016, the nonavalent HPV (9vHPV) vaccine replaced the 4vHPV vaccine [1]. The program was also extended that same year to boys in grade 4 and men who have sex with men (MSM) aged up to 26 years [1, 2]. Data showed that a mixed schedule comprising 1 dose of 9vHPV vaccine and 1 dose of bivalent (2vHPV) vaccine is safe and elicits a high immunogenic response against HPV-16 and HPV-18 [3, 4], the 2 genotypes responsible for the majority of HPV-attributable cancers [5], HPV-16 being the most oncogenic genotype [6, 7]. Considering data since 2011 showing the efficacy of a 1-dose schedule [8–12] and a lower unit price for the 2vHPV vaccine compared to the 9vHPV vaccine, the Quebec Immunisation Committee recommended the use of a mixed schedule-the second dose (2vHPV) being considered a “safety net” against HPV-associated cancers in case further data with 1-dose vaccine would not support previously published data [9]. In 2018, Quebec became the first jurisdiction to adopt a mixed schedule for vaccinating 9- to 17-year-old girls and boys. This approach made it possible to offer HPV vaccination to additional birth cohorts (boys in grade 9) [1, 4]. Considering that the mixed schedule (9vHPV + 2vHPV) was innovative, an evaluation plan was judged important, starting with a prevalence study among nonvaccinees to estimate herd immunity.

Indeed, when devising an evaluation strategy for new vaccination schedules, the presence of a herd effect must be taken into consideration. A study conducted in Quebec 5 years after the implementation of the initial vaccination program targeting girls showed a prevalence of 1% for 4vHPV vaccine–targeted genotypes in 17- to 19-year-old women [13]. Data from other jurisdictions support the decline in prevalence of HPV vaccine genotypes in unvaccinated men following the implementation of female vaccination through herd protection [14–16]. In Quebec, HPV vaccination coverage (VC) is high among young women, with 90% having received at least 1 dose when measured at 15 years old [17]. A modeling study suggests that a sustained VC of 80% or more among girls and boys could eliminate vaccine-targeted HPV genotypes through herd immunity [18], implying that the 4vHPV vaccine–targeted genotypes may no longer be circulating in Quebec, including among unvaccinated same-age individuals as those targeted by the public vaccination program. Therefore, it was deemed relevant to measure the prevalence of HPV genotypes among unvaccinated individuals before designing a study measuring direct vaccine efficacy comparing vaccinees and nonvaccinees or vaccinees having received different schedules, including the mixed schedule. In our case, the female population could not be targeted for this study, since most females aged 9–28 years had already been vaccinated against HPV, achieving a high VC (>80%) [1, 17]. Likewise, girls and boys vaccinated using the mixed schedule in grade 4 were too young at the time of this study to be potentially infected with HPV infection. Thus, only boys in grade 9 aged 14–15 years also eligible for vaccination with the mixed schedule since September 2018 [19] were of age to be at risk of infection and could be targeted for the evaluation of the schedule change. The precise VC among 16- to 20-year-old men in 2020–2022 is unknown but was estimated to be very low, since this cohort was not eligible for the public vaccination program. A 2014 study conducted among parents of 9- to 16-year-old boys living in Quebec (16–23 years old in 2021) revealed that 1% of boys were vaccinated and 5% of parents intended to vaccinate (with costs) their boy against HPV [20].

This cross-sectional study aimed to measure the prevalence of HPV infection among unvaccinated sexually active 16- to 20-year-old men and to explore factors associated with HPV detection.

## MATERIALS AND METHODS

### Study Population

From February to September 2020, male subjects aged 16–20 years were recruited in colleges and vocational schools located in the Greater Quebec City area. Recruitment paused due to the coronavirus disease 2019 (COVID-19) pandemic and resumed in April 2021, expanding to schools in other regions of Quebec and online. Eligibility criteria included understanding French or English and providing informed consent. Participants, from this convenience sample, were excluded if they reported no sexual contact in their lifetime, had received 1 or more doses of an HPV vaccine, or were immunocompromised or took immunosuppressive medication at the time of recruitment. Ethical approval for this study was granted by the Research Ethics Board of the Centre Hospitalier Universitaire de Québec-Université Laval (#2020–5037), and the study is registered at ClinicalTrials.gov (NCT04297670).

### Study Procedures

After signing the consent form, subjects were asked to complete an online questionnaire on their sociodemographic characteristics, lifestyle habits, sexual practices, sexual health, and HPV vaccination status. Participants’ self-reported vaccination status (unvaccinated) was verified using the Quebec Vaccination Registry and the vaccination record. Only unvaccinated participants were eligible for this study.

Participants self-collected a penile swab for HPV testing as reported elsewhere [21]. In summary, participants were instructed to rub the entire penis shaft at least 15 times with a flocked swab, avoiding all contact with the pubic hair. An instructional video was provided and participants were offered a free 9vHPV vaccine dose after their participation in the study.

### HPV Testing

All samples underwent HPV detection and genotyping using the Anyplex II HPV28 Detection assay (Seegene Inc). This test allows for the detection of 28 oncogenic, possibly oncogenic, and nononcogenic HPV genotypes (6/11/16/18/26/31/33/35/39/40/42/43/44/45/51/52/53/54/56/58/59/61/66/68/69/70/73/82) [22]. Swab samples were agitated in 1 mL of 20 mmol/L Tris buffer, pH of 8.3, and DNA was purified using the MasterPure protocol. β-globin gene coamplification screened for the presence of inhibitors or degraded or inadequate quantities of human DNA. Samples negative for β-globin were considered inadequate for polymerase chain reaction (PCR) analysis. Negative and positive controls provided in the HPV28 kit were included in each HPV28 run, as suggested by the manufacturer. Precautions to avoid contamination were effective at all times. Samples positive for ß-globin and negative for HPV were considered HPV negative. For all HPV analyses, the HPV risk categories are based on the classification established by the International Agency for Research on Cancer [23]. HPV-16/18/31/33/35/39/45/51/52/56/58/59/68 are considered to be high-risk oncogenic genotypes and detectable by the Anyplex II test. Genotypes 6 and 11 are also detectable as well as other low-risk genotypes presented in Supplementary Table 2.

### Statistical Analyses

Descriptive analyses were conducted to determine the prevalence of HPV overall and each HPV genotype separately, as well as the distribution of key demographic, sexual, and health characteristics. Fisher exact test was used to compare participant characteristics according to HPV detection. Univariate and multivariate analyses identified participant characteristics associated with HPV presence. Three mutually exclusive categories were defined regarding the participants' type of sexual partners: women only, men only, and both genders. All independent variables, representing potential factors associated with HPV detection according to scientific literature, were included in the initial logistic regression model and reduced by backward elimination to obtain the final model. Unadjusted and adjusted odds ratios (ORs), 95% confidence intervals (CIs), and *P* values (2-tailed) were reported as measures of association. If participants provided a “not sure/prefer not to answer” response to a question, it was treated as missing data and excluded from univariate and multivariate analyses. An α level of .05 (5%) was used to establish statistical significance. All statistical analyses were conducted using SAS software version 9.4.

## RESULTS

### Number and Characteristics of Participants Recruited

**Figure 1.**
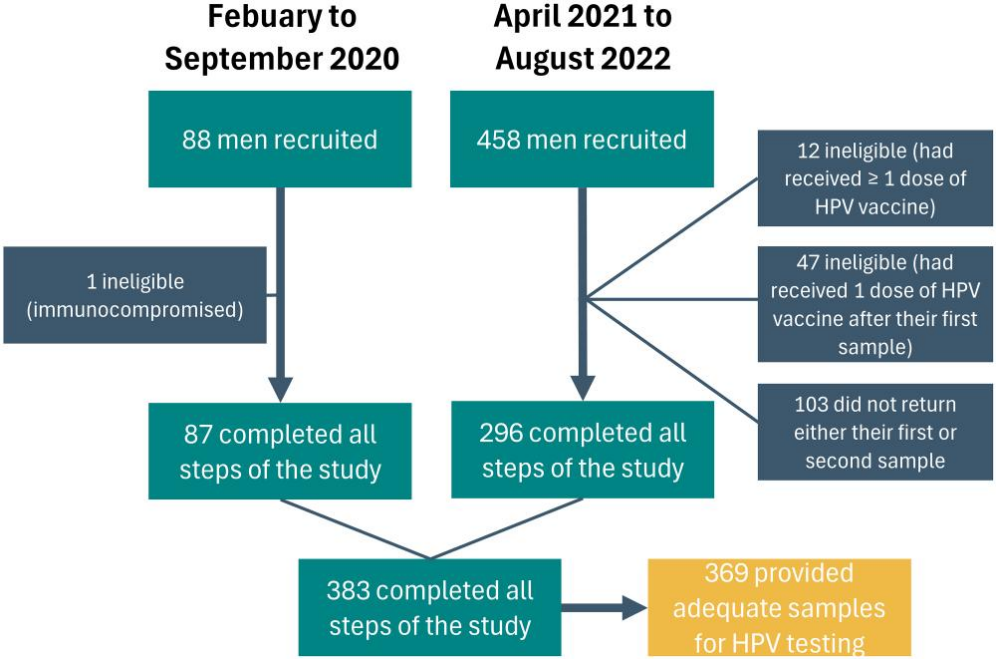
Participants recruited during the 2 recruitment periods. Abbreviation: HPV, human papillomavirus.

Between February and September 2020, 88 men were recruited in colleges and vocational schools in the Greater Quebec City area (Figure 1). Another 458 men were recruited online or in colleges across the province of Quebec from April 2021 to August 2022. Of those 546 men aged 16–20 years, 12 were excluded for receiving an HPV vaccine prior to the study and 1 for being immunocompromised. Additionally, 190 samples were lost during transportation to the laboratory. Of those whose samples were lost, 47 had already received an HPV vaccine following the collection of their first penile sample, making them ineligible to participate again. A total of 143 participants were therefore asked to provide a second sample during the course of the study. Overall, 103 participants did not return either their first or second (repeat) sample after giving their consent to participate in the study. Of the 383 participants who completed all study procedures, 14 were excluded because their sample was inadequate for PCR analysis, leaving 369 participants for the analysis.

Demographic and health behavioral characteristics of participants are shown in Table 1. The mean age of men included in this study was 18.9 years (median, 19 years). Most participants were born in Canada, were French-Canadian, lived in the Greater Quebec City or Central Quebec areas, and had a post–high school education.

**Table 1.** Demographic and Lifestyle Characteristics as Reported by Study Participants (N = 369)

Variable	No.	(%)
Age, y		
17^a^	27	(7.3)
18	93	(25.2)
19	148	(40.1)
20	101	(27.4)
Main occupation		
Vocational school student^b^	83	(22.5)
Post–high school education student	275	(74.5)
Worker	11	(3.0)
Country of birth		
Canada	334	(90.5)
Other	35	(9.5)
Area of residence		
Greater Montreal	34	(9.2)
Greater Quebec City	238	(64.5)
Central Quebec	68	(18.4)
Western Quebec	4	(1.1)
Eastern Quebec	25	(6.8)
Self-reported ethnicity^c^		
French-Canadian	306	(82.9)
Other	58	(15.7)
Smoking status (≥100 cigarettes in lifetime)^d^		
Yes	40	(10.8)
No	310	(84.0)
Vaping (daily)^e^		
Yes	75	(20.3)
No	293	(79.4)

^a^Includes 3 participants aged 16 years.

^b^Includes 3 participants in high school and 1 in adult school.

^c^Not sure/prefer not to answer responses were provided by 5 (1.4%) participants.

^d^Not sure/prefer not to answer responses were provided by 19 (5.1%) participants.

^e^Not sure/prefer not to answer responses were provided by 1 (0.3%) participant.

### Sexual Habits of Participants

The sexual behavior characteristics of participants are shown in Table 2 (as reported in the questionnaire). Most men reported sexual contacts with female partners. When categorized by type of partners (based on questionnaire answers), 83% (307/369) of participants had sexual contact with women only, while 8% (30/369) and 8% (30/369) had sexual contact with men only and with partners of both genders, respectively. Two participants (1%) did not disclose the gender of their partners and therefore could not be assigned to 1 of the 3 categories of sexual partner types. The mean and median numbers of lifetime partners were 4.2 and 3 for those who had sex with women only, 5.7 and 3 for those who had sexual contact with men only, and 8.7 and 5 for those with partners of both genders.

**Table 2.** Lifetime Sexual Behaviors as Reported by Study Participants

Characteristic	With Female Partner		With Male Partner	
	No.	(%)	No.	(%)
Sexual contacts^a^	n = 369		n = 369	
Yes^b^	335	(90.8)	60	(16.3)
Lifetime No. of partners	308	(91.9)	52	(86.7)
1–2	138	(44.8)	26	(50.0)
3–4	75	(24.3)	14	(29.9)
≥5	95	(30.8)	12	(23.1)
Unknown	27	(8.1)	8	(13.3)
No	30	(8.1)	307	(83.2)
Penetrative sex (vaginal or anal)	n = 335		n = 60	
Yes	329	(98.2)	38	(63.3)
Lifetime No. of partners	299	(90.9)	30	(78.9)
1–2	157	(52.5)	20	(66.7)
3–4	64	(21.4)	6	(20.0)
≥5	78	(26.1)	4	(13.3)
Unknown	30	(9.1)	8	(21.0)
No	6	(1.8)	22	(36.7)
Age at first penetrative sex, y	n = 329		n = 38	
≤15	106	(32.2)	9	(23.7)
16	101	(30.7)	8	(21.0)
17–20	122	(37.1)	21	(55.3)

	With Any Partner (Female or Male)	
	n = 369	(%)
Condom use or other latex barrier during sexual contact^c^		
Always	75	(20.3)
Sometimes	184	(49.9)
Never	96	(26.0)
Use of condom during last penetrative sex^d^		
Yes	141	(38.2)
No	222	(60.2)
Previously informed of an STI^e^ diagnosis (by nurse/doctor)^f^		
Yes	10	(2.7)
No	354	(95.9)

Abbreviation: STI, sexually transmitted infection.

^a^Two participants did not disclose the gender of their partners.

^b^Not sure/prefer not to answer responses were provided by 4 (1.1%; with female partner) and 2 (0.5%; with male partner) participants.

^c^Not sure/prefer not to answer responses were provided by 14 (3.8%) participants.

^d^Not sure/prefer not to answer responses were provided by 6 (1.6%) participants.

^e^The following STIs were inquired about: genital warts (condylomas), genital herpes, chlamydia, gonorrhea, syphilis, hepatitis B, and human immunodeficiency virus.

^f^Not sure/prefer not to answer responses were provided by 5 (1.4%) participants.

Almost all participants (98%) reporting sexual contact with female partners had penetrative sexual intercourse with them. This percentage was lower (63%) among participants reporting male partners. The average age of first penetrative sexual intercourse among those who had sexual contact with women only, men only, and both genders was 16.1, 16.8, and 15.2 years, respectively.

Among all participants, few (n = 10) had already received a sexually transmitted infection (STI) diagnosis; only 1 reported having had genital warts (condylomas). Of the 10 participants, 2 were MSM (*P* ≥ .05). Sexual behavior characteristics over the 12 months prior to completion of the questionnaire are available in Supplementary Table 1.

### HPV Prevalence

The prevalence of penile HPV genotypes detected among participants is shown in Table 3. The prevalence of any of the 28 HPV genotypes tested was 18%. The prevalence of the 13 high-risk types was 13%. The number of HPV genotypes per positive sample varied from 1 to 9. Among infected participants, 49% had >1 HPV genotype infections. The combined prevalence of 4vHPV vaccine–targeted genotypes was very low, with only 1 HPV-11 detected in 1 participant and 1 HPV-18 detected in another, both in MSM. The only participant who tested positive for HPV-11 reported no history of genital warts. The combined prevalence of 9vHPV vaccine–targeted genotypes was 5%. Positivity results by HPV genotype are available in Supplementary Tables 2 and 3.

**Table 3.** Prevalence of Penile Human Papillomavirus Genotypes Among Unvaccinated Male Subjects

HPV Infection Type	No.	(%)	95% CI
Any of 28 HPV genotypes	68	(18.4)	14.6–22.8
Any of 13 high-risk genotypes	47	(12.7)	9.5–16.6
Any of 15 low-risk genotypes	48	(13.0)	9.8–16.9
Any quadrivalent vaccine genotypes	2	(0.5)	.1–1.9
Any nonavalent vaccine genotypes	18	(4.9)	2.9–7.6
HPV-6/11	1	(0.3)	.0–1.5
HPV-16/18	1	(0.3)	.0–1.5

Abbreviations: CI, confidence interval; HPV, human papillomavirus.

### Characteristics Associated With the Detection of HPV

**Figure 2.**
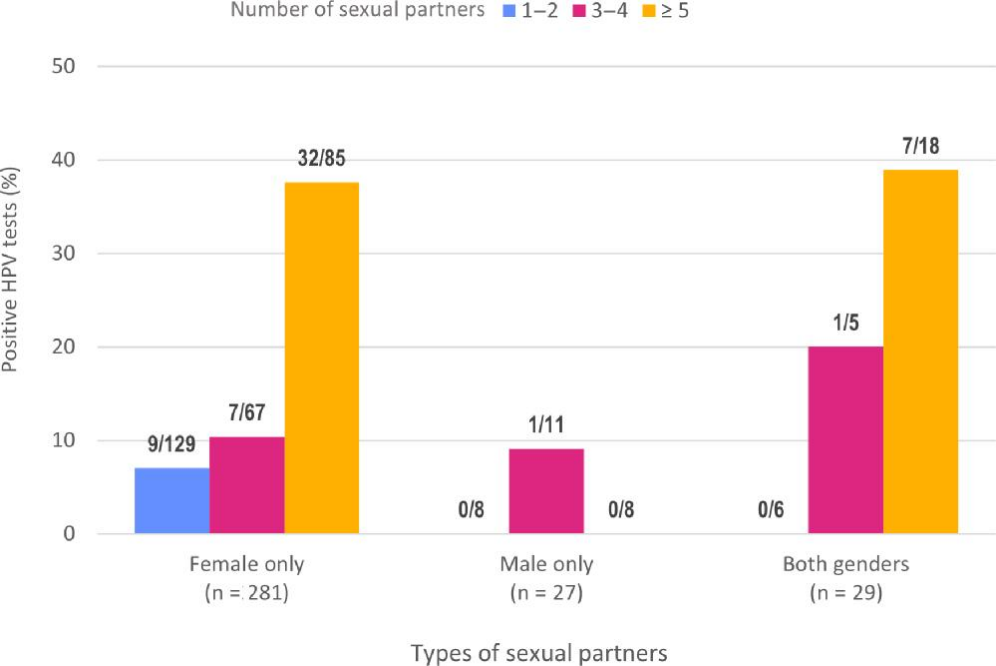
Proportion and number of participants who tested positive for at least 1 human papillomavirus (HPV) genotype according to type and number of lifetime sexual partners. Participants who did not declare their number of partners were excluded.

Characteristics associated with the detection of at least 1 HPV genotype included having reported a greater number of lifetime partners (Figure 2), a history of STIs (60% vs 17%), smoking ≥100 cigarettes in one's lifetime (35% vs 16%), not always using a condom (sometimes or never 21% vs always 9%) and being older (22% among 19- to 20-year-olds, compared with 12% among 16- to 18-year-olds).

Table 4 presents the univariate and multivariate analysis based on 311 participants for whom we had no missing data with regard to analyzed variables. The final model was adjusted for confounding variables (condom use and type of partners), as the adjusted ORs varied by >10% without this adjustment. The multivariate analysis identified age, number of lifetime sexual partners, and history of STIs as being independently associated with HPV detection. Sensitivity analyses were carried out to account for participants who had missing data for the number of sexual partners, but these analyses did not alter the conclusions of the multivariate model presented in Table 4. Two models were analyzed where missing data participants were included (1) in the “5 or more partners” category, since their prevalence of at least 1 HPV genotype was very similar to the prevalence of participants who reported having had 5 or more partners in their lifetime, and (2) as an additional category (Supplementary Table 4).

**Table 4.** Human Papillomavirus–Associated Characteristics in Univariate and Multivariate Analyses

Characteristic	HPV Positive	HPV Negative	Univariate Analysis			Multivariate Analysis^a^		
	n = 51	n = 260	OR	95% CI	*P* Value	OR	95% CI	*P* Value
Age								
16–18 y	10	95	reference			reference		
19–20 y	41	165	2.36	1.13–4.93	.0222	2.43	1.10–5.37	.0288
No. of partners (lifetime)								
1–4	18	195	reference			reference		
≥5	33	65	5.50	2.90–10.42	<.0001	4.73	2.39–9.37	<.0001
Type of partners								
Female only	43	216	reference			…	…	
Male only	1	25	0.20	.03–1.52	.1204	…	…	
Both genders	7	19	1.85	.73–4.67	.1928	…	…	
Condom use						…	…	
Always	6	60	reference			…	…	
Sometimes	34	125	2.72	1.08–6.83	.0332	…	…	
Never	11	75	1.47	.51–4.20	.4751	…	…	
History of STIs						…	…	
No	46	257	reference			reference		
Yes	5	3	9.31	2.15–40.31	.0028	4.82	1.01–22.88	.0479
Smoking ≥100 cigarettes (lifetime)								
No	42	240	reference			…	…	
Yes	9	20	2.57	1.10–6.03	.0299	…	…	
Region of residence^b^								
Where 1-dose HPV VC is ≥95%	40	205	reference			…	…	
Where 1-dose HPV VC is <95%	11	55	1.03	.49–2.13	.9469	…	…	
Self-reported ethnicity								
French-Canadian	45	220	reference			…	…	
Other	6	40	0.73	.29–1.83	.5072	…	…	

Abbreviations: CI, confidence interval; HPV, human papillomavirus; OR, odds ratio; STI, sexually transmitted infection; VC, vaccination coverage.

^a^Model adjusted for condom use and type of partners.

^b^The region of residence was classified according to the VC among girls at age 15 (at least 1 dose) in each health region. Source: [17].

## DISCUSSION

We observed an overall HPV prevalence of 18.4% (95% CI, 14.6%–22.8%) and a prevalence of 4vHPV vaccine–targeted genotypes (HPV-6/11/16/18) of 0.5% (95% CI, .1%–1.9%) among sexually active unvaccinated 16- to 20-year-old men. The most probable explanation for the low 4vHPV vaccine prevalence observed in our study (0.5%) stems from the herd immunity effect conferred by HPV vaccination of same-age girls as the male subjects included in this study, via the school-based vaccination program implemented since 2008. As of 2019 (just before the start of the study), all female residents of Quebec aged ≤28 years had been eligible for free HPV vaccination and 90% had received at least 1 dose by age 15 [24]. Both participants harboring a 4vHPV vaccine–targeted genotype reported sexual contact with other men and consequently could have benefited less from the indirect protection conferred by the immunization of girls. The higher prevalence observed for 9vHPV vaccine–targeted genotypes (4.9% [95% CI, 2.9%–7.6%]) is not surprising, since girls were vaccinated with the 4vHPV vaccine up until 2016 (9vHPV vaccine thereafter). Vaccination of boys remains justified to offer direct protection to all men before the onset of sexual activity, particularly for men who will have sex with men [2, 25].

Our findings are similar to those of 2 Australian studies conducted between 2014 and 2017 in unvaccinated men aged 16–20 years, where both showed an overall HPV prevalence of approximately 20% and 3% of 4vHPV vaccine prevalence [15, 16]. In 2017, VC of at least 1 dose in Australia was 88.9% among 15-year-old girls [26], which is similar to the VC reported in Quebec [17, 19, 27]. A systematic review of literature published before the implementation of HPV vaccination programs worldwide showed an overall HPV prevalence between 26% and 65% among young (<30 years old) general (low-risk) North American populations [28]. The prevalence of HPV-16 and -18 was estimated at 5%–11% and 2%–3%, respectively, at this time. The prevalence of high-risk HPV genotypes, including all genotypes targeted by the 9vHPV vaccine, was 20%–29%. In a study conducted in Quebec (Montreal region) prior to the implementation of the vaccination program among 263 young couples (median and mean, 22 and 22.7, respectively, for men and 21 and 21.2 for women), the prevalence of at least 1 HPV genotype detected in male partners was 56%. Genotype HPV-16 was the most common genotype, detected in 16.4% of males [29, 30].

Whereas participants who reported a higher number of sexual partners over their lifetime were proportionally likelier to test positive for at least 1 HPV genotype, no 4vHPV vaccine–targeted HPV genotypes were detected in men reporting sexual contacts with female partners only. This finding is consistent with a previous study conducted among 17- to 29-year-old women in Quebec 5 years after the HPV vaccination program implementation, which showed an association between the number of sexual partners and the detection of nonvaccine HPV genotypes and no association for vaccine-targeted HPV genotypes among vaccinated women [13]. The number of lifetime sexual partners is well known to be the strongest risk factor for HPV infection among men and women [31–33]. HPV vaccination is highly effective to avoid vaccine-targeted HPV genotype infections regardless of the number of lifetime sexual partners.

MSM in our study reported a higher number of lifetime partners, which aligns with prior research [34]. The percentage of MSM in our study was also higher (16.3%) than expected when compared to provincial and national surveys [35–38]. However, a US study that estimated the percentage of MSM across different states showed that estimates varied widely (ie, estimates ranged from 1.5% in Wyoming to 18.5% in California) [39]. A systematic review explored the choice of method for estimating MSM populations and found that studies using surveillance data obtained the highest estimates whereas those using populational survey data obtained the lowest [40]. Penetrative sexual contacts were also less frequent in MSM in our study (63.3%), which aligns with a US study conducted among 14- to 17-year-old MSM in which 62.1% reported having anal sex among those having declared sexual contacts and reported using MSM-specific apps to meet partners [41].

Our study has many strengths. Only 3.7% of penile swabs collected by participants were inadequate for HPV analysis. This rate is lower than that reported in other studies among men using a similar methodology, where 10%–15% of samples were considered unsuitable for testing [15, 16]. The use of an instructional video, a flocked swab, guidelines to rub the entire penis shaft at least 15 times, and a laboratory with extensive experience conducting HPV testing for research purposes [7, 13, 22, 29–31] most likely contributed to the higher proportion of valid samples for HPV detection in the current study. Participants' self-reported vaccination status (unvaccinated) was verified through the Quebec Vaccination Registry and the vaccination record.

The study also has limitations. First, the loss of 190 samples before reaching the laboratory required many participants to collect and send a repeat sample. However, those who had received an HPV vaccine dose after their first sample (n = 47) were ineligible to participate again, and many failed to return a new sample. Second, the use of an online recruitment method and the inclusion of different educational institutions across the province did result in the recruitment of participants from most regions of Quebec, but the majority of participants (64.5%) were residents of 1 region (Greater Quebec City area). Updated data shows that this region's VC is among the highest in the whole province [27]. Other regions, such as Greater Montreal, where VC is lower [27], accounted for a smaller proportion of the overall sample. This could have resulted in an underestimation of 4vHPV vaccination prevalence within the study population if herd effect is smaller in areas with lower VC. The underestimation is probably of limited magnitude, considering that (1) VC comprising at least 1 dose exceeds 80% in all Quebec regions [17], when measured among 15-year-old girls; (2) mathematical modeling studies have shown that ≥80% of VC can eliminate vaccine-targeted genotypes [18]; and (3) 4vHPV vaccine–targeted genotypes detected in the current study were all detected among MSM (minority). Third, as the recruitment and data collection for 282 participants (76.4%) took place during the COVID-19 pandemic, the preventive measures could have affected the number of sexual contacts of our subjects. However, overall 10.9% of participants reported having had 5 or more partners in the previous 12 months compared to 7.1% in a provincial survey conducted among a similar age group just prior to the pandemic [42]. Fourth, due to the COVID-19 pandemic, recruitment was interrupted and restarted with a revised method. However, despite being a convenience sample, the overall study population was similar to the general Quebec population of the same age in terms of smoking status, condom use, and history of STIs [35], all of which are factors associated with the prevalence of HPV infections [32, 33, 43, 44]. It is reassuring that 18% of our sample had at least 1 HPV type detected and 5% of the 5 supplementary targeted types in the 9vHPV vaccine, but not in the 4vHPV vaccine, the latter being the one received by same-age girls as our participants. These prevalences are expected for 16- to 20-year-old men [14–16] and reinforce that our sample was sexually active, harbored non-vaccine-targeted HPV types, and could have tested positive for vaccine-targeted types if present. The combined prevalence for HPV-33/45/52/58 among similar-aged men conducted before HPV vaccination program implementation was 4.6% [30], compared to our observed prevalence of 4.8%, indicating similar HPV acquisition risk and supporting the study's external validity.

## CONCLUSIONS

The very low prevalence of the 4vHPV vaccine–targeted genotypes among the unvaccinated male population of the same age as women who were eligible for vaccination suggests a strong herd immunity. Evaluation of schedule changes, including the recent change to a single 9vHPV vaccine dose for all 9- to 20-year-olds in Quebec [45–48], will need to take this finding into account. In jurisdictions with high VC, such as Quebec, it will be impossible to measure direct vaccine efficacy comparing vaccinees and nonvaccinees since both of these groups are protected by the HPV vaccine program. Surveillance through the detection of HPV genotypes targeted by the vaccines (especially genotypes 16/18) via cervical cancer screening activities using HPV testing will be a valid and efficient strategy to quickly detect a change in the protection offered by the vaccination program. Overall, this study supports the population effectiveness of HPV vaccination in jurisdictions such as Quebec where VC among youth is high.

## Supplementary Material

jiaf094_Supplementary_Data

## Supplementary Data

Supplementary materials are available at *The Journal of Infectious Diseases* online (http://jid.oxfordjournals.org/). Supplementary materials consist of data provided by the author that are published to benefit the reader. The posted materials are not copyedited. The contents of all supplementary data are the sole responsibility of the authors. Questions or messages regarding errors should be addressed to the author.

## Notes

***Acknowledgments.*** We thank all the participants and all the staff involved in the study.

***Financial support.*** This study was funded by the Quebec Ministry of Health and Social Services.
