# Supplementary material for: Prevalence of Human Papillomavirus Genotypes in Unvaccinated 16- to 20-Year-Old Men in Quebec, Canada
Source: J Infect Dis. 2025 Feb 21;232(2):e203–12. doi: 10.1093/infdis/jiaf094 (PMC12349958; doi:10.1093/infdis/jiaf094)
Supplement: jiaf094_Supplementary_Data [file jiaf094_supplementary_data.docx]

**Supplementary material**

Supplementary Table 1. Sexual behaviors in the past 12 months as reported by study participants

|  | With female partner | | With male partner | |
| --- | --- | --- | --- | --- |
| Sexual contacts | **n = 335** | **%** | **n = 60** | **%** |
| Yes | 313 | 93.4 | 42 | 70.0 |
| *Number of partners in the past 12 months* | *291* | *93.0* | *39* | *92.9* |
| *1-2 partners* | *213* | *73.2* | *25* | *64.1* |
| *3-4 partners* | *48* | *16.5* | *9* | *23.1* |
| *≥ 5 partners* | *30* | *10.3* | *5* | *12.8* |
| *Unknown number of partners* | *22* | *7.0* | *3* | *5.0* |
| No | 22 | 6.6 | 18 | 30.0 |
| Penetrative sex (vaginal or anal)^a^ | **n = 329** | **%** | **n = 38** | **%** |
| Yes | 311 | 94.5 | 28 | 73.7 |
| *Number of partners in the past 12 months* | *280* | *90.0* | *26* | *92.9* |
| *1-2 partners* | *215* | *76.8* | *19* | *73.1* |
| *3-4 partners* | *40* | *14.3* | *5* | *19.2* |
| *≥ 5 partners* | *25* | *8.9* | *2* | *7.7* |
| *Unknown number of partners* | *31* | *10.0* | *2* | *5.3* |
| No | 17 | 5.2 | 10 | 26.3 |

^a^ Not sure/Prefer not to answer responses were provided by 1 (0.3%) participant with female partner.

Supplementary Table 2. Positivity Results by HPV Genotype for Low-Risk HPVs

| Low-risk HPV genotypes | Number of positive HPV | Positivity (%) |
| --- | --- | --- |
|  | **n = 369** | |
| HPV-6^a^ | 0 | 0.0 |
| HPV-11^a^ | 1 | 0.3 |
| HPV-26 | 1 | 0.3 |
| HPV-40 | 3 | 0.8 |
| HPV-42 | 8 | 2.2 |
| HPV-43 | 11 | 3.0 |
| HPV-44 | 1 | 0.3 |
| HPV-53 | 11 | 3.0 |
| HPV-54 | 9 | 2.4 |
| HPV-61 | 0 | 0.0 |
| HPV-66 | 13 | 3.5 |
| HPV-69 | 0 | 0.0 |
| HPV-70 | 0 | 0.0 |
| HPV-73 | 12 | 3.3 |
| HPV-82 | 7 | 1.9 |
| Total ^b^ | **77** | **20.9** |

^a^ 9vHPV genotypes
^b^ The presence of each individual genotype detected is detailed here, including when several genotypes were detected in the same sample. Table 3 of the main manuscript shows the proportion of samples in which at least one genotype was detected.

Supplementary Table 3. Positivity Results by HPV Genotype for High-Risk HPVs

| High-risk HPV genotypes | Number of positive HPV | Positivity (%) |
| --- | --- | --- |
|  | **n = 369** | |
| HPV-16^a^ | 0 | 0.0 |
| HPV-18^a^ | 1 | 0.3 |
| HPV-31^a^ | 0 | 0.0 |
| HPV-33^a^ | 2 | 0.5 |
| HPV-35 | 1 | 0.3 |
| HPV-39 | 8 | 2.2 |
| HPV-45^a^ | 3 | 0.8 |
| HPV-51 | 17 | 4.6 |
| HPV-52^a^ | 6 | 1.6 |
| HPV-56 | 7 | 1.9 |
| HPV-58^a^ | 7 | 1.9 |
| HPV-59 | 17 | 4.6 |
| HPV-68 | 3 | 0.8 |
| Total ^b^ | **72** | **19.5** |

^a^ 9vHPV genotypes
^b^ The presence of each individual genotype detected is detailed here, including when several genotypes were detected in the same sample. Table 3 of the main manuscript shows the proportion of samples in which at least one genotype was detected.

Supplementary Table 4. Sensitivity multivariate analyses with missing data for the number of partners variable

| **Characteristics** | **HPV positive** | **HPV negative** | **n=330** | | | |
| --- | --- | --- | --- | --- | --- | --- |
|  | **n=57** | **n=273** | **OR** | **95% CI** | | **p value** |
|  | Sensitivity multivariate analysis 1^a,b^ | | | | | |
| **Age** | | | | | | |
| 16-18 years | 10 | 99 | reference | | |  |
| 19-20 years | 47 | 174 | 2.70 | 1.24 | 5.92 | 0.0127 |
| **Number of partners (lifetime)** | | | | | | |
| 1 to 4 | 18 | 195 | reference | | |  |
| 5 or more | 39 | 78 | 4.72 | 2.46 | 9.03 | <0.0001 |
| **History of STIs** | | | | | | |
| No | 52 | 270 | reference | | |  |
| Yes | 5 | 3 | 4.74 | 1.00 | 22.47 | 0.0502 |
|  | Sensitivity multivariate analysis 2^a,c^ | | | | | |
| **Age** | | | | | | |
| 16-18 years | 10 | 99 | reference | | |  |
| 19-20 years | 47 | 174 | 2.69 | 1.23 | 5.90 | 0.0134 |
| **Number of partners (lifetime)** | | | | | | |
| 1 to 4 | 18 | 195 | reference | | |  |
| 5 or more | 33 | 65 | 4.65 | 2.35 | 9.18 | <0.0001 |
| Missing | 6 | 13 | 5.05 | 1.64 | 15.53 | 0.0047 |
| **History of STIs** | | | | | | |
| No | 52 | 270 | reference | | |  |
| Yes | 5 | 3 | 4.77 | 1.00 | 22.67 | 0.0494 |

^a^ Model adjusted for condom use and type of partners.

^b^ Missing data participants (number of partners variable) were included in the "5 or more partners" category.

^c^ Missing data participants (number of partners variable) were included as an additional category.
